# Analysis of couch position tolerance limits to detect mistakes in patient setup

**DOI:** 10.1120/jacmp.v10i4.2864

**Published:** 2009-10-29

**Authors:** Scott W. Hadley, James M. Balter, Kwok L. Lam

**Affiliations:** ^1^ Department of Radiation Oncology Physics The University of Michigan Medical School Ann Arbor MI USA

**Keywords:** patient safety, treatment errors, record and verify, immobilization

## Abstract

This work investigates the use of the tolerance limits on the treatment couch position to detect mistakes in patient positioning and warn users of possible treatment errors. Computer controlled radiotherapy systems use the position of the treatment couch as a surrogate for patient position, and a tolerance limit is applied against a planned position. When the couch is out of tolerance, a warning is sent to a user to indicate a possible mistake in setup. A tight tolerance may catch all positioning mistakes while at the same time sending too many warnings; a loose tolerance will not catch all mistakes. We developed a statistical model of the absolute position for the three translational axes of the couch. The couch position for any fraction is considered a random variable $x_{i}$. The ideal planned couch position $x_{p}$ is unknown before a patient starts treatment and must be estimated from the daily positions of $x_{i}$. As such, $x_{p}$ is also a random variable. The tolerance, *tol*, is applied to the difference between the daily and planned position, $d_{i}=x_{i}-x_{p}$. The $d_{i}$ is a linear combination of random variables and therefore the density of $d_{i}$ is the convolution of distributions of $x_{i}$ and $x_{p}$. Tolerance limits are based on the standard deviation of $d_{i}$ such that couch positions that are more than two standard deviations away are considered out of tolerance. Using this framework, we investigated two methods of setting $x_{p}$ and tolerance limits. The first, called first day acquire (FDA), is to take couch position on the first day as the planned position. The second is to use the cumulative average (CumA) over previous fractions as the planned position. The standard deviation of $d_{i}$ shrinks as more samples are used to determine $x_{p}$ and, as a result, the tolerance limit shrinks as a function of fraction number when a CumA technique is used. The metrics of sensitivity and specificity were used to characterize the performance of the two methods to correctly identify a couch position as in‐ or out‐of‐tolerance. These two methods were tested using simulated and real patient data. Five clinical sites with different indexed immobilization were tested. These were whole brain, head and neck, breast, thorax, and prostate. Analysis of the head and neck data shows that it is reasonable to model the daily couch position as a random variable in this treatment site. Using an average couch position for $x_{p}$ increased the sensitivity of the couch interlock and reduced the chances of acquiring a couch position that was a statistical outlier. Analysis of variation in couch position for different sites allowed the tolerance limit to be set specifically for a site and immobilization device. The CumA technique was able to increase the sensitivity of detecting out‐of‐tolerance positions while shrinking tolerance limits for a treatment course. Making better use of the software interlock on the couch positions could have a positive impact on patient safety and reduce mistakes in treatment delivery.

PACS number: 87.55.Ne, 87.55.Qr, 87.55.tg, 87.55.tm

## I. INTRODUCTION

Computer controlled radiotherapy and record and verify systems (R&V) were introduced to allow for complex treatments and increase the safety of radiation delivery.^(^ ^1^ ^–^ ^7^ ^)^ It was found that these systems improve patient safety and reduce treatment errors when used properly.^(^ ^8^ ^–^ ^14^ ^)^ The work presented here focuses on the use of one aspect of the R&V system related to the software interlock applied to the position of the treatment couch. This software interlock is used to provide a level of automatic oversight to the patient setup by applying a tolerance limit to a baseline position of the treatment couch. When used properly this interlock may aid in warning users of potential mistakes in the patient setup.

We use the term “mistake” to mean that the process to setup the patient has a failure that puts the patient in the wrong position for treatment. We use this term to distinguish this type of error from the more common “setup error” or “positioning error”, which is often used to mean the systematic and random geometric displacement of a target from its planned position. Some examples of setup mistakes are: setting the incorrect source to surface distance, setting up to the wrong mark on a patient with multiple isocenters, not applying a necessary shift from a setup mark or even using the wrong treatment plan as a result of a patient identification error. Correct setups would be those that used the correct information and in which all setup instructions were followed correctly.

This aspect of the R&V systems uses the treatment couch location as a surrogate for patient position and the software interlock acts as a binary classifier to determine correct and incorrect setups. The software will allow treatment to continue if the couch position is within a preset tolerance limit, or it can trigger a software interlock if the position is out of tolerance. In this work, we investigated statistical methods to determine baseline values and tolerance limits for the position of the treatment couch for a patient's course. The goal is to make the best use of this software system to improve patient safety.

X‐ray imaging is the main method to correct patient positioning errors. Much work has been done on online and offline strategies for correcting systematic and random errors in patient position.^(^ ^15^ ^–^ ^18^ ^)^ Imaging may or may not be used every day depending on what strategy is used. There is also a possibility that the interpretation of the image is incorrect and a mistake in patient position is undetected. Some radiation therapy treatments don't use X‐ray imaging at all, and thus don't benefit from these methods. Errors in the patient setup may be present when imaging is not used. In these situations the R&V system software interlock on the couch position may be the only electronic and automatic check of patient setup. Given that R&V systems are installed and in use in the vast majority of radiation therapy clinics, careful analysis of their use seems warranted.

Much work has been done to improve immobilization of patients for many different treatment sites.^(^ ^19^ ^–^ ^26^ ^)^ When an immobilization device is indexed to the table, it attaches rigidly in the same place for each fraction and, theoretically, improves the coupling between the digital position readout of the couch and the patient's placement with respect to the isocenter. This would improve the ability of the R&V system to detect mistakes in setup.

Previous work in radiation therapy investigated the use of the R&V system to understand variations in machine parameters and their possible impact on patient safety. Podmaniczky et al.^(4)^ recognized the utility of the R&V system to collect data on patient setup, and analyzed the variations that exist in the axes of the treatment machine. They used statistical analysis of recorded histories to set tolerance limits for patient setups. Patton et al.^(11)^ compiled a review of errors and determined, among other things, that indexed immobilization along with couch position tolerance limits was an important part of achieving correct patient setup. They also recognized the interplay between couch tolerance limits and usability by therapists to detect incorrect patient positioning. Klein et al.^(27)^ used different tolerance limits based on indexed immobilization and the treatment type.

In this work, we critically analyze the use of the couch digital position readout as a surrogate for the patient position. We investigate the question of how the couch digital can be used as part of the quality assurance process to eliminate gross mistakes in treatments. Couch positions from patient treatment records are used to determine the variation in couch locations for different treatment sites and immobilization devices. The ability of indexed immobilization to reduce variation in digital readout of the treatment couch is investigated by calculating standard deviations from mean couch positions for a course of treatment. We develop a statistical analysis of patient data in order to set planned couch positions that improve the ability of the tolerance limit to detect out‐of‐tolerance patient setups. To test our methods, we employ the metrics of sensitivity and specificity to quantify the ability of different techniques to correctly classify couch positions as being in or out of tolerance. This type of quantitative analysis may allow users to adjust the tolerance limits to control the expected number of warnings sent to users.

## II. MATERIALS AND METHODS

We consider the position of the couch on any given fraction to be an independent random variable drawn from the probability density function *p(x)* where *x* is one of the translational axes of the treatment couch. The density function *p(x)* will have both correct and incorrect patient setups. The actual form *of p(x)* will depend on the isocenter location, treatment site, positioning errors, and the indexed immobilization that is used for the treatment. The presence of systematic positioning errors is not explicitly dealt with in the following method. Instead, we rely on the patient positioning and imaging protocols to deal with possible systematic errors in position that may be present in a patient setup.

We take a statistical approach to the problem of setting planned couch values and tolerance limits for patient treatments. Let the random variable *x* represent one axis of the couch, which is sampled from the density function p(μ,σ) with a mean *μ* and standard deviation *s.* We assume that the couch position *x* for each fraction is independent and identically distributed. The ideal planned couch position, *x* must be *μ*. We assume that couch positions that are far from *μ* indicate a possible mistaken setup. The variance σ indicates the ability of the immobilization device to reposition the patient on the table, as well as random error that may exist in the patient positioning. Current treatment delivery software allows for a planned value *x* to be set for each field, while the tolerance limit is set from a smaller set of tables that cannot be patient‐specific. The tolerance limit is applied symmetrically around the planned position.

### A. Average Couch Position

For any given patient treatment, we assume that *μ* and σ are not known prior to treatment. The planned couch position, $x_{p}$, must be estimated from positions obtained for each fraction. Because we assume that the couch position from any fraction is a random variable, then the estimated planned couch position is also a random variable. The ideal unknown planned couch position *μ* can be estimated using an average over previous fractions during a patient's course of treatment,

(1) $$x_{p}=\frac{1}{n}\sum_{i=1}^{n}x_{i},$$

where $x_{i}$ is the couch position on the $i^{\text{th}}$ fraction and *n* is the number of previous fractions. The estimation of the average position, $x_{p}$, improves with each fraction such that the standard deviation of $x_{p}$ is $\sigma /\sqrt{n}$. $x_{p}$ is a random variable with density function $p(\mu ,\;\sigma /\sqrt{n})$.

### B. Couch deviations from a planned position

The tolerance limit must be compared to the difference from the planned position and the couch position for that fraction, $d_{i}=x_{i}-x_{p}$. Because $d_{i}$ is the difference of two random variables, the density function of $d_{i}$ is the convolution of *p(μ, σ)* with $p(\mu ,\sigma /\sqrt{n})$ with zero mean and the standard deviation, $\sigma_{d},$, taken in quadrature.

(2) $$\sigma_{d}=\sqrt{\sigma^{2}+\frac{\sigma^{2}}{n}}=\sigma \sqrt{\frac{n+1}{n}}$$

The density function of differences $d_{i}$ always has a standard deviation equal to or larger than the couch positions $x_{i}$ and is a function of the fraction number *n.* Equation 2 provides a method for shrinking the tolerance limit as more couch positions are averaged together, and the estimate of $x_{p}$ improves if the assumption that $x_{i}$ is independent and identically distributed holds for a given patient treatment. This assumption can be violated by clinically relevant situations – such as adjustments to patient setup (due to weight loss that requires the couch to move systematically to compensate), or the use of an imaging protocol that adjusts position on days when imaging is used but not on other days.

### C. Strategies for setting planned couch values and tolerance limits

We attempt to balance two goals when setting tolerance limits. One is to set tolerance limits with tight geometric constraints, and the other is to set the limit at a level that will not send too many false warnings to an operator that may be a clinical burden and lead him/her to mistrust the warning. We chose to set the tolerance limit to trigger a warning on 5% of the fractions treated on average.

Two strategies for setting planned couch values and tolerance limits were investigated. The first is based on what current treatment control software allows for in a clinical setting. For example, our software system, Varis (Varian Medical Systems, Palo Alto CA), allows for each field to have its own planned couch position with a tolerance table attached to it. A limited number of tolerance tables can be defined and used in the R&V system. Based on these limitations, the couch position on the first fraction is used as the planned position for the remaining fractions. The tolerance limit, *tol*, is set to twice the population standard deviation of the differences $d_{i}$ based on Eq. 2, where $n=1,\text{tol}=2\sigma_{\text{pop}}\sqrt{2}$ With this tolerance, couch positions should be identified as out of tolerance 4.5% of the fractions on average. We call this method the First Day Acquire (FDA) method.

The second strategy uses averaging of couch positions to improve the planned couch value and bring it closer to the unknown average position for a patient. We call this the cumulative average (CumA) method. Once again the tolerance limit is set equal to twice $\sigma_{d}$ but we use Eq. 2 to shrink the limit as the number of samples in the average increase.

(3) $$\text{tol}(n)=2\sigma_{d}=2\sigma_{\text{pop}}\sqrt{\frac{n}{n-1}}$$

Using this method, the tolerance limit becomes a function of the fraction number when *n* is greater than 1 and will shrink as more patient specific information is derived. For the first fraction, the difference $d_{i}$ is always zero. Therefore, the tolerance limit has no meaning. There is no ability to determine if there is a mistake in the setup based on the couch position on the first fraction.

### D. Performance evaluation

The software interlock is acting like a binary classifier to make a decision about setups that may or may not have errors. Specificity and sensitivity are often used to characterize the performance of binary classifiers. To evaluate the performance of these two methods, we compare specificity and sensitivity estimates. For the clinical data, couch positions that were more than $2\sigma_{\text{pop}}\sqrt{2}$ away from the patient's mean overall fractions were chosen to represent positions that were out of tolerance. This defined the ground truth data to determine which couch positions were in or out of tolerance. When the FDA and CumA techniques were applied to the datasets, their ability to detect out‐of‐tolerance couch positions was tested against the ground truth data. Specificity and sensitivity were calculated from the number of true positives (NTP), false positives (NFP), true negatives (NTN), and false negatives (NFN). The percentage of couch positions that were determined as out of tolerance was also calculated.

(4) $$\begin{array}{ll} \text{sen}\text{sit}\text{ivi}ty=\frac{\text{NTP}}{\text{NTP}+\text{NFN}}\;\;\;\;\;\;\;\; & \text{spe}\text{cif}\text{ici}ty=\frac{\text{NTN}}{\text{NTN}+\text{NFP}} \end{array}$$

### E. Testing using clinical and artificial data

These two methods were tested using clinical and artificial data. The simulation data was used to test the two methods under ideal conditions and served as a basis to compare to the performance using clinical data. For any given dataset, the couch positions that were more than two standard deviations away from the mean were considered out of tolerance.

Clinical patient data was obtained under an institutional review board approved retrospective study. Couch coordinates from patients treated in our department were used to test the two methods of setting planned couch positions and tolerance limits. The position of the couch was taken from the treatment field history, and represents the actual couch position for treatment after patient setup and any image guidance. Patients were stratified by treatment site and type of indexed immobilization device. The treatment sites were whole brain, head and neck (H&N) intensity‐modulated radiation therapy (IMRT), breast, thorax and prostate IMRT. The immobilization devices used were the Sinmed Posifix for whole brain and H&N setups, Posiboard for breast setups, and the Posirest (all by Civco Medical Solutions, Kalona, Iowa) for thorax setups. All immobilization devices were indexed to the table top. Prostate patients did not use an immobilization device and were not indexed to the treatment table. The IMRT treatments used daily imaging to correct for systematic and random errors. Prostate patients were aligned to implanted gold markers and H&N cases were aligned to bony anatomy. Non‐IMRT cases used an imaging protocol to detect systematic patient positioning errors within the first five fractions of treatment and then used weekly imaging thereafter. Table 1 summarizes the number of patients and fractions for the five groups.

The head and neck IMRT data provided the best opportunity to analyze the statistical nature of the digital couch position due to the use of good indexed immobilization and daily image guidance. The mean of each patient for each couch axis was subtracted for the raw couch coordinates. Statistical analysis was done on this dataset to characterize the variation between patients by calculating each patient's standard deviation for each axis of the treatment couch. A population standard deviation was calculated for each treatment fraction to study trends in variation over the course of treatment.

For each of the five sites, the population standard deviation, $\sigma_{\text{pop}}$, was calculated and used to determine the couch positions that were greater than $2\sigma_{\text{pop}}$ from the patient's mean overall fractions. This was used as ground truth data to which the FDA and CumA techniques were compared. For each of five sites and two techniques, the specificity and sensitivity were calculated across all fractions.

**Table 1:** Summary of clinical data used to test methods.

	Number of Patients	Average Number Of Fractions	Total Fractions
Whole Brain	36	11	409
Head & Neck	51	34	1728
Breast	79	25	2010
Thorax	87	21	1803
Prostate	68	40	2719

For the simulated data, a single translation of the treatment couch was modeled as a zero mean Gaussian distribution with a standard deviation of 1 cm. A 35‐fraction treatment was simulated for 1,000,000 patients. The couch position for each fraction was determined using a random number generator, and the FDA and CumA techniques were applied to the same dataset. The sensitivity, specificity, and percentage of out‐of‐tolerance warnings were calculated for the two methods as a function of fraction number as well as overall fractions.

## III. RESULTS

### A. Clinical data

**Figure 1:**
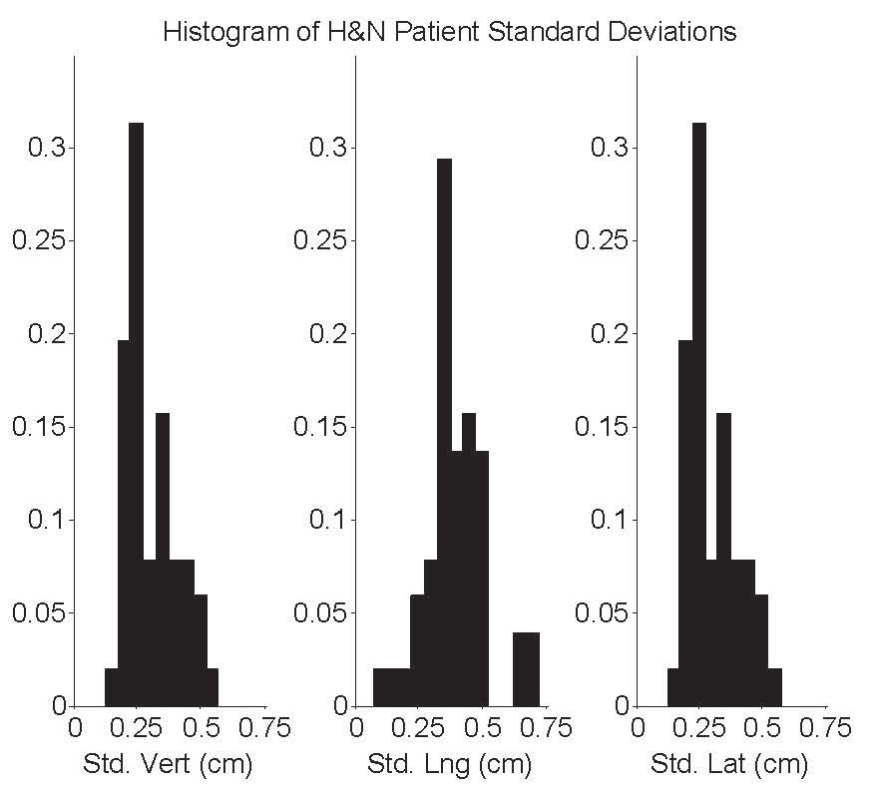
Histograms of the standard deviation of each H&N patient's digital couch coordinates over the course of their treatment.

**Figure 2:**
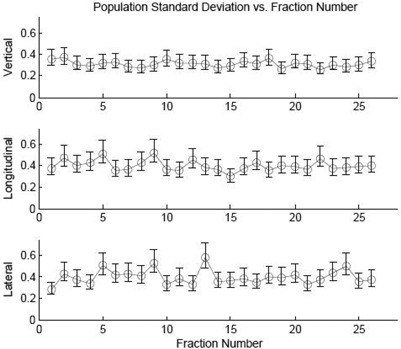
Graph of the population standard deviation of H&N treatment couch positions on a treatment fraction basis with corresponding 95% confidence intervals. All patients had at least 26 fractions treated. No trend can be seen in the data to indicate that any fraction has more or less variation than another.

The standard deviation for each H&N patient was calculated and plotted as a histogram in Fig. 1. The population standard deviation within a fraction is graphed in Fig. 2. For this well‐indexed and immobilized treatment site with daily image guidance there is no indication that the table position on any given fraction is more correct than any other.

**Figure 3:**
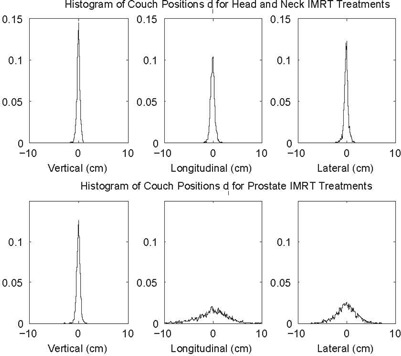
Histogram of couch positions after the average of each patient has been subtracted, $d_{i}=x_{i}-\mu$. The H&N IMRT patients are shown in the top row; the prostate IMRT patients in the bottom row. Both of these treatment sites used daily image guidance to remove systematic and random errors in the target position. The large differences between the two sites are due to the high level of index immobilization used in H&N and no immobilization used for prostate cases.

Population standard deviations and tolerance limits for the two methods for the three translational axes of the couch for the five treatment sites are shown in Table 2. The tolerance limit for the FDA technique is calculated from Eq. 3, using n=1. For the CumA technique, the tolerance limit is a function of the fraction number. The value in Table 2 is the limit near the end of a typical number of fractions for that body site. Histograms of couch positions after the patient average has been subtracted can be seen in Fig. 3. The two sites shown are H&N and prostate IMRT. These two sites used daily image guidance to reduce systematic and random setup errors for each fraction. Despite image guidance, variation still exists in the position of the treatment couch during a course of treatment.

**Table 2:** Population standard deviation and tolerance limits for each couch translation and treatment site.

		Vertical	Long.	Lateral
	$\sigma_{\text{pop}}\;(\text{cm})$	0.32	0.34	0.27
Whole Brain	FDA *tol* (cm)	0.9	1.0	0.8
	CumAtol(n=15)(cm)	0.7	0.7	0.6
	$\sigma_{\text{pop}}\;(\text{cm})$	0.32	0.42	0.42
Head & Neck	FDA *tol* (cm)	0.9	1.2	1.2
	CumAtol(n=35)(cm)	0.7	0.9	0.9
	$\sigma_{\text{pop}}\;(\text{cm})$	0.35	0.87	1.30
Breast	FDA *tol* (cm)	1.0	2.5	3.7
	CumAtol(n=25)(cm)	0.7	1.8	2.7
	$\sigma_{\text{pop}}\;(\text{cm})$	0.32	1.20	1.33
Thorax	FDA *tol* (cm)	0.9	3.4	3.8
	CumAtol(n=20)(cm)	0.7	2.5	2.7
	$\sigma_{\text{pop}}\;(\text{cm})$	0.36	3.08	1.99
Prostate	FDA *tol* (cm)	1.0	8.7	5.6
	CumA tol(n=42)(cm)	0.7	6.2	4.0

Table of standard deviations shows digital readouts from the mean for the five different body sites and the corresponding tolerance limit determined using Eq. 3. The CumA tolerance limit is a function of fraction number and value above is the tolerance at the end of a typical number of fractions for the give body site.

**Figure 4:**
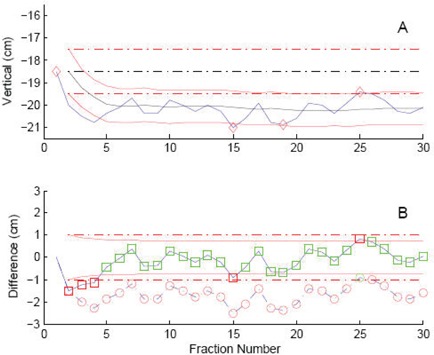
Graphs of table vertical coordinates and differences for a selected patient. Graph A shows the absolute coordinate of the couch (blue) where the couch positions more than $2\sigma_{\text{pop}}$ from the mean are marked with a red diamond and are considered to be out of tolerance. The planned couch position is shown in black, with the FDA method as a broken line and the CumA method as a solid line. The tolerance limit is show as a red broken line. Graph B shows the same data, but plots the difference from the planned values. The CumA technique is plotted to indicate positions marked as out of tolerance (red squares) and within tolerance (green squares). The FDA technique is plotted with circles.

An example of the FDA and CumA technique applied to the vertical couch axis of a selected breast patient's daily couch position is shown in Fig. 4. This case was chosen because the first fraction had the largest deviation from the average for the vertical position of the couch. For the FDA method, each day except for one is out of tolerance. The CumA technique incorporates the planned value each day to the average of the previous fractions. The two methods disagree with respect to the couch being in or out of tolerance for most fractions.

Sensitivity and specificity for the FDA and CumA techniques are summarized in Tables 3 and 4. The results of the simulation data are included for comparison. Results for the five sites generally show that sensitivity increased when the CumA technique was used. The exception is the whole brain dataset where sensitivity decreased. This dataset was investigated and it was found that the first fraction represented the mean better than the cumulative average up to about the 6th fraction treated. This may be a result of the specifics of the patient setup where the marks on the immobilization mask are set to lasers, and setup imaging is only used on the first day and then every 5th fraction thereafter.

The percentage of treatments marked as out of tolerance are presented in Table 5. The Table shows the percentage of fractions that were determined to be out of tolerance by the two techniques. Additionally, the percentage of fractions that have any of the axes as out of tolerance is presented. The cumulative average technique reduced the number of out‐of‐tolerance warnings for each individual axis, as well as overall for any axis. For four of the five sites studied, the out‐of‐tolerance warning occurred on about 10% of all fractions treated. This would mean that, on average, fractions treated would have an axis out of tolerance for one out of every 10 fractions treated.

**Table 3:** Sensitivity values for the two methods and all five treatment sites.

		Sensitivity for Vertical	Sensitivity for Long.	Sensitivity for Lateral
Whole Brain	FDA	0.60	0.38	0.50
	CumA	0.20	0.31	0.44
Head & Neck	FDA	0.34	0.47	0.48
	CumA	0.56	0.60	0.73
Breast	FDA	0.41	0.45	0.36
	CumA	0.56	0.56	0.58
Thorax	FDA	0.29	0.32	0.46
	CumA	0.47	0.53	0.59
Prostate	FDA	0.50	0.34	0.39
	CumA	0.60	0.54	0.67
Simulated Data	FDA		0.32	
	CumA		0.72	

**Table 4:** Specificity values for the two methods and five treatment sites.

		Specificity for Vertical	Specificity for Long.	Specificity for Lateral
Whole Brain	FDA	0.92	0.98	0.97
	CumA	1.00	0.99	1.00
Head & Neck	FDA	0.97	0.97	0.99
	CumA	0.99	0.99	1.00
Breast	FDA	0.89	0.97	0.98
	CumA	1.00	0.99	1.00
Thorax	FDA	0.90	0.97	0.95
	CumA	0.98	1.00	1.00
Prostate	FDA	0.95	0.96	0.97
	CumA	0.99	0.99	0.97
Simulated Data	FDA		0.97	
	CumA		0.99	

**Table 5:** Table of percentage of fractions that are out of tolerance for the two methods.

		% Out of Tol. Vertical	% Out of Tol. Long.	% Out of Tol. Lateral	% Out of Tol. Any Axis
Whole Brain	FDA	9.54%	3.42%	4.89%%	15.65%
	CumA	0.98%	1.71%	1.96%	4.40%
Head & Neck	FDA	5.15%	5.21%	3.59%	12.44%
	CumA	4.11%	3.59%	4.46%	10.76%
Breast	FDA	11.99%	4.98%	4.13%	19.25%
	CumA	3.08%	3.03%	3.38%	8.71%
Thorax	FDA	10.65%	4.16%	6.66%	19.91%
	CumA	4.16%	2.72%	3.27%	9.54%
Prostate	FDA	6.66%	5.44%	4.78%	16.15%
	CumA	3.20%	3.79%	3.64%	10.26%

### B. Artificial data

**Figure 5:**
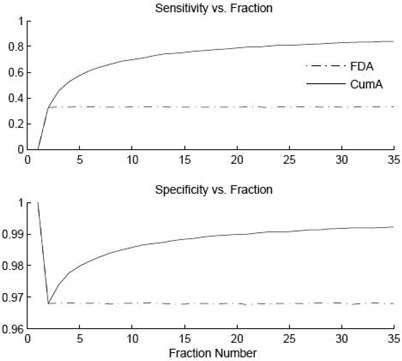
Graph of the sensitivity and specificity for the FDA (broken line) and CumA (solid line) techniques as a function of fraction number for simulated data. The two techniques are equivalent for the first two fractions. The CumA technique shows improvement in the ability to detect which couch positions are out of tolerance.

A graph of the sensitivity and specificity of the simulation data as a function of fraction number is shown in Fig. 5. It can be seen that on the first fraction, when no information is known about where the couch should be, the tolerance limit is insensitive to mistakes in patient setup. By the second fraction, the first day acquire (FDA) method has set the planned couch position, and the sensitivity has moved to 0.33 and is unchanged for the remaining fractions. The cumulative average technique, CumA, is identical to the FDA method for the first two fractions. With the third fraction, the cumulative average increased the sensitivity of the couch tolerance limit to detect out‐of‐tolerance couch positions. By the last fraction, 35, the CumA technique had increased the sensitivity to 0.84, while the FDA method was at 0.33.

The sensitivity over all 35 fractions for the FDA and CumA techniques was 0.32 and 0.72, respectively. The specificity for FDA and CumA was 0.97 and 0.99, respectively. The specificity for both methods started off at 1.0 and then dropped to 0.97 on the second fraction where the FDA remains for all 35 fractions. The CumA method improves the specificity over the 35 fractions to 0.99. Both techniques maintained a rate of out‐of‐tolerance warnings of 4.6% for each fraction after the first. The CumA technique had the advantage of a shrinking tolerance level while maintaining a constant rate of out‐of‐tolerance warnings.

## IV. DISCUSSION

Record and verify systems were created to increase the accuracy and safety of radiation therapy treatments. Much of the work on quality assurance and R&V systems has been to verify that the correct data is entered into the system. While many users report on the quality assurance of the R&V systems, few have focused on how the R&V system participates in the quality assurance of treatment delivery. In this work, we focused on a software interlock applied to the daily couch position that is intended to exert control over possible mistakes made in the patient setup process. In order to understand how well this system can reduce possible mistakes in patient setup, we analyzed the variation that exists in the system by using recorded treatment histories from patients treated in our department.

The results in Table 2 clearly indicate that different sites require different tolerance limits if an operator wants to maintain the same rate of warning to the users. If one tolerance limit were set for all sites, then some sites would trigger more interlocks and receive additional attention while another site may not benefit at all. The policy in our department is that, when a couch parameter is out of tolerance, a procedure is triggered such that the radiation therapists review the essential parts of the patient setup to check for any mistakes. Additional X‐ray imaging may be performed to verify the positioning. If a tolerance were set too tight, therapists would be investigating a large number of patient setups that have no mistakes. This would be a burden on a busy clinic for only limited improvement in the quality of patient setups. Knowing the actual variation that exists in couch positions for a given immobilization device would allow one to set tolerance limits to balance the needs of clinical efficiency with good oversight of patient setup. How to set those limits is a policy issue that should involve discussion with physicians and administrators, and should consider the ability of the couch digital readout – in conjunction with the immobilization device – to provide good information about possible mistakes in patient setup.

The results of the H&N data validate the idea that the position of the treatment couch can be considered a random variable with an unknown average and standard deviation. There is no evidence in the data to suggest that the position of the couch on any given fraction is better or more correct than for another fraction. Given that, it would seem reasonable to use averaging to estimate a better baseline value for the couch position for the software interlock. The result of estimating a better baseline value for the software interlock makes the system more sensitive to positions that are out of tolerance. Using an average position also guards against acquiring a table position that is an outlier in the distribution of table positions even though the target position may be correct. The simulation experiments showed large increase in sensitivity due to averaging and shrinking tolerance limits. This could be an advantage to users of the system for it increases their trust that the system is providing accurate information.

The sensitivities reported in Table 3 show that the interlock system is much less sensitive than the ideal (1.0). How sensitive the system is depends on its tolerance limit. In this work, we used Eq. 3 to set tolerance limits that were statistically consistent with the underlying data. For example, the tolerance limit on the lateral couch position for prostate patients is over 4.5 times larger than for a well‐indexed head and neck patient. If a more sensitive system is desired, one either needs to improve the quality of the immobilized indexing or shrink the tolerance limit. Shrinking the tolerance limit without improving the indexed immobilization would decrease the specificity. Despite the relativity loose tolerances in Table 2 and the low sensitivities in Table 3, Table 5 shows that anywhere from 4.5% to 20% of fractions will have an out‐of‐tolerance warning, with most sites triggering an interlock on over 10% of fractions treated.

Much work has been done on the management of systematic and random errors in target position for conformal radiation therapy. The results of serial imaging have been used to determine the average positioning error and to apply a shift to future treatments to reduce systematic errors. Random errors in target position are often dealt with by using daily imaging to correct the target position. It is not the purpose of the software interlock on the couch readout to provide oversight of the target positioning, but rather provide some level of quality assurance against mistakes in positioning. In this work, we ignored the presence of systematic errors in the target position. If a systematic error were found and corrected by use of an imaging protocol, that same shift could be applied to the average couch position to produce a baseline value that incorporates the systematic error.

## V. CONCLUSIONS

In this work, we investigated a new method to set planned couch positions and adapt the tolerance limits based on updated information about the patient setup. It was shown that by adapting the planned position and tolerance limit based on data obtained during treatment, the R&V system could make better use of the software interlock. Increases in patient safety may be possible by modifying R&V systems to take into account possible errors.

## ACKNOWLEDGEMENTS

This work was supported by NIH grant P01CA59827.
